# The influence of motivational climate on the physical activity adherence among junior high school students: The mediating effect of achievement goal orientation

**DOI:** 10.1371/journal.pone.0315831

**Published:** 2024-12-19

**Authors:** Ning Yang, Haiying Quan, Zimeng Guo

**Affiliations:** School of Physical Education, Liaoning Normal University, Dalian, China; Southwest University, CHINA

## Abstract

In this study, which was based on the theories of motivational climate and achievement goal orientation, an experimental intervention was conducted on 82 junior high school students using the Motivational Climate Scale (MCSYS), Task and Ego Orientation in Sport Questionnaire (TEOSQ), and Physical Activity Persistence Scale. The aim was to explore the influence of different motivational climates in physical education classes on the physical activity adherence among junior high school students and examine the mediating role of achievement goal orientation. The results revealed the following significant findings: Firstly, there was a noteworthy positive correlation between the physical activity adherence among junior high school students, the motivational climate, and achievement goal orientation. Additionally, it was discovered that achievement goal orientation partially mediated the relationship between the motivational climate and the physical activity adherence among junior high school students. Secondly, it was observed that the effectiveness of improving the physical activity adherence among junior high school students increased with the duration of the experimental intervention. Lastly, it was found that the mastery climate group had a more pronounced effect on improving the physical activity adherence among junior high school students when compared to the performance climate group. These findings suggest that creating a motivational climate in physical education classes has the potential to modify the achievement goal orientation of junior high school students, subsequently enhancing their consistency in engaging in physical activity.

## 1. Introduction

Physical activity adherence refers to the regularity, continuity, and long-term nature of an individual’s engagement in physical activities, maintaining a particular movement or behavior in a stable state [1]. The measurement of physical activity adherence not only involves quantitative aspects but can also be approached from the psychological perspective of an individual’s internal psychological manifestations and effort in participating in physical activity activities [2]. With the transformation and development of the education industry in China, enhancing the participation and habits of adolescents in physical activity and improving their physical fitness has become a key focus of educational reform. As vital participants in the realization of the “Healthy China” strategy, junior high school students play a crucial role. Guiding adolescents at this stage to develop good physical activity participation and adherence habits can effectively lay a solid foundation for their physical and mental health. The junior high school stage is a transitional phase of receiving school education, and school sports play a pivotal role in cultivating students’ good adherence to physical activity behaviors [3]. Since entering the new era, China’s educational endeavours have been oriented towards reducing the academic burden on students and promoting their healthy and all-round development, both physically and mentally. Against the background of educational transformation, all sectors of society are concerned about the physical and mental health development of young people, and school education is also constantly establishing the educational concept of ‘health first’, so that students can enhance their physical fitness, improve their personality and enjoy themselves in physical exercise. The junior high school stage is the articulation and transition stage between primary school initiation and senior high school education, and students at this stage are in an important period of forming their outlook on life and values. Therefore, under the guidance of national policies and measures, the teaching of school physical education classroom can help the physical fitness and health level of junior high school students to continuously step up to a new level, and guide young people at this stage to form a good participation in exercise and persistence in exercise, which will make junior high school students’ physical exercise show the situation of “participation is easy, and persistence is effective”, which has an important significance to the promotion of the construction of “Healthy China”. It is of great significance to promote the construction of ‘Healthy China’.

Numerous studies have demonstrated that motivational climate is a crucial environmental factor influencing the formation of individuals’ adherence to physical activity. Motivational climate refers to the dominant goal orientation individuals possess in specific achievement situations, which is determined by the interaction between individuals and their environment. It represents an environmental goal structure that emerges in specific contexts [4]. During the educational process in classrooms, different classroom climates can be created by employing the five dimensions of the TARGET theory within the framework of motivational climate theory: task, authority, recognition and evaluation, grouping, and timeframe. By incorporating collaborative and motivational teaching methods, a motivational climate can be established in physical education classes, thereby enhancing students’ adherence to physical activities [5]. As research progresses, it becomes evident that the influence of motivational climate on the consistency of physical activity adherence may also be related to achievement goal orientations.

Achievement goal orientation emphasizes individuals’ perceptions of themselves and the task, as well as their understanding of the importance of achievement. Based on their perception of goal characteristics, individuals make cognitive representations that correspond to the environmental structure of goal orientation [6]. Existing studies have indicated that the contextual characteristics displayed in the classroom can influence individuals’ goal orientation choices. Achievement goal orientation reflects individuals’ cognitive tendencies toward achievement situations, with the mastery and performance climates within the motivational climate significantly predicting two dimensions of achievement goal orientation: task orientation and ego orientation [7]. Additionally, an individual’s tendency toward achievement goal orientation as a psychological factor directly influences their motivation to engage in physical activity. Research has shown that task goal orientation has a positive impact on the consistency of physical activity, while ego goal orientation to some extent decreases individuals’ adherence to physical activity behaviors [8]. By considering achievement goal orientation as a moderating variable to observe the relationship between motivational climate and achievement goal orientation, investigations have found a correlation between physical activity adherence and achievement goal orientation and motivational climate. It is worth noting that the enhancement effect of ego goal orientation on individuals’ willingness for physical activity continuity is not significant. Therefore, to improve individuals’ adherence to physical activity, it is important not only to consider the motivational climate in the classroom but also to pay close attention to individuals’ achievement goal orientation [9]. Regarding the influence of ego and task orientations on individuals, different scholars have proposed different viewpoints. Currently, research has not reached a unanimous conclusion on the negative impact of ego goal orientation on individuals. Multiple research results suggest that the influence of ego goal orientation on individuals is complex in terms of its extent and effects. Therefore, it is crucial to further examine its role and determine the conditions under which it has positive or negative effects [10]. In terms of the duration of interventions involving motivational climate and achievement goal orientation on physical activity adherence, numerous scholars have conducted experiments over varying numbers of weeks. However, existing research has indicated that the improvement effect of achievement goal orientation on physical activity adherence increases with the duration of physical activity [11]. To further investigate changes in physical activity adherence under different intervention durations, measurements of physical activity adherence were performed at the 4th, 8th, and 12th weeks of the experiment, allowing for observation of changes in physical activity adherence with varying intervention durations.

In conclusion, guided by the motivational climate theory, achievement goal orientation theory, and the TARGET theory, research has brought to light the impact mechanism of motivational climate on the adherence of physical activity among junior high school students from the perspective of creating a motivational climate in physical education classrooms. Furthermore, the study also explores the varying scenarios of physical activity adherence among junior high school students under different intervention durations. The study posits the following hypotheses: (1) The motivational climate can predict junior high school students’ physical activity adherence and their individual achievement goal orientation. (2) Achievement goal orientation mediates between the motivational climate and physical activity adherence. (3) The improvement effect on the physical activity adherence of the mastery climate group of junior high school students is superior to that of the performance climate group. (4) The effectiveness of improving physical activity adherence varies under different intervention durations, and the longer the intervention duration, the greater the improvement in physical activity adherence.

## 2. Methodology

### 2.1 Research object

The research employed the pwr package in the R programming language to conduct an analysis for the required sample size. Assuming a large effect size (*d* = 0.80) and a statistical power of*1-β* = 0.8, with *α* = 0.05, it was determined that at least 26 participants per group were needed. The study utilized a cluster sampling method, selecting 82 participants from two classes in the second year of junior high at the 49th Middle School in Dalian. Among the selected participants, there were 46 boys and 36 girls, with an average age of 13.39 years. The chosen sample size aligns with the G-power calculated sample size, and none of the participants were involved in any extracurricular sports interest groups. This research was approved by the Ethics Committee of Liaoning Normal University and was found to be in compliance with the requirements of ethics, with the Ethics Review No. LL2023072. Consent for this study was obtained from the guardians participating in the study. The experiment time is: 07/09/2023-04/12/2023.

### 2.2 Research instruments

#### 2.2.1 Motivational climate questionnaire

The measurement of motivational climate utilized the *Motivational Climate Scale* (MCSYS), which was adapted by Ronald [12] from the *Perceived Motivational Climate in Sport Questionnaire-II* (PMCSQ-2) developed by Duda. The revised scale is more suitable for students aged 9–14 and consists of two dimensions: mastery orientation climate and performance orientation climate. The scale comprises 12 items, and scores were calculated separately for each dimension. A Likert 5-point scoring method was employed, ranging from “completely inconsistent” to “completely consistent”, with a total score range of 1–5. Higher scores indicate a stronger motivational climate established by physical education teachers. The Cronbach’s α coefficient for this questionnaire in this study was 0.927.

#### 2.2.2 Achievement goal orientation questionnaire

The measurement of achievement goal orientation utilized the *Task and Ego Orientation in Sport Questionnaire* (TEOSQ). This questionnaire consists of 13 items that assess task goal orientation and ego goal orientation. Likert 5-point scoring method was used, with response options ranging from “completely inconsistent” to “completely consistent”, corresponding to scores ranging from 0 to 4. The total score is calculated by summing individual item scores, indicating the level of goal orientation. In this study, the Cronbach’s α coefficient for this questionnaire was 0.945.

#### 2.2.3 Physical activity adherence questionnaire

The measurement of physical activity adherence utilized the *Physical Activity Adherence Scale* developed by Wang [2]. This scale consists of 14 items, which were categorized into three dimensions: effort investment, emotional experience, and behavioral habits. A Likert 5-point scoring method was used, ranging from “completely disagree” to “completely agree”, with scores ranging from 1 to 5. Higher total scores on this scale indicate a higher level of adherence to physical activity behavior. In this study, the Cronbach’s α coefficient for this questionnaire was 0.920.

### 2.3 Data collection and analysis

The study employed SPSS 27.0 and the PROCESS plugin for data processing. Pre-tests and post-tests for motivational climate, physical activity adherence among junior high school students, and achievement goal orientation were conducted before and after the formal experiment to assess the homogeneity of each group before testing and the intervention’s effects. Additionally, physical activity adherence was measured at the 4th, 8th, and 12th weeks of the intervention to assess the improvement effect of physical activity adherence at different intervention durations.

### 2.4 Experimental design and test procedures

#### 2.4.1 Experimental design

The study used a one-way between subject design, with the independent variable being the physical education classroom motivational climate, including mastery motivational climate and achievement motivational climate at two levels, the dependent variable being junior high school students’ adherence to physical activity, and the mediating variable being achievement goal orientation, including task goal orientation and self goal orientation. The contents of physical education teaching in both experimental classes were arranged according to the teaching schedule of physical education courses in the tested grades, and the specific operation of classroom motivational climate creation was carried out according to the designed classroom motivational climate implementation plan.

#### 2.4.2 Test procedures

The study was conducted with the consent of the students, teachers and guardians of the students. The experimental intervention was conducted for a total of 12 weeks, excluding national holidays, three times a week. Measurement questionnaires were distributed before and after the experimental intervention, and pre- and post-test data collection was conducted to compare the effects of the experimental intervention. At the same time, questionnaires were distributed in the 4th, 8th and 12th weeks of the experiment to collect data three times to examine the changes in adherence to physical activity under different lengths of the experimental intervention.

## 3. Results and analysis

### 3.1 Test for common method bias

To avoid the presence of common method bias in the experimental data collected through intervention and questionnaire distribution, this study employed procedural controls and conducted a Harman’s single-factor test to address the potential common method bias effect in the experiment. In terms of procedural controls, mature and validated measurement tools were selected for data collection, emphasizing that the survey data was for research purposes only and had no right or wrong answers. The anonymity and confidentiality of the data were repeatedly emphasized, and data were collected on-site through paper-based questionnaires. Regarding the Harman’s single-factor test, an exploratory factor analysis (EFA) was conducted on all items of the scales. The results extracted 12 factors with eigenvalues greater than 1, and the variance explained by the first factor was 29.93%, which is less than 40%. This finding indicates that the common method bias in this study falls within an acceptable range.

### 3.2 Descriptive statistics and correlation analysis (redone using the initial dataset)

#### 3.2.1 Correlation analysis of variables

The existence of a two-by-two correlation between the variables is a prerequisite for the mediation effect test, therefore, the study started with descriptive statistics and correlation analyses of the collected data. The results of the correlation analysis of the research variables indicate a significant positive correlation between motivational climate and physical activity adherence among junior high school students (*P*<0.01). Furthermore, there exists a significant positive correlation between motivational climate and achievement goal orientation (*P*<0.05). Additionally, each dimension of achievement goal orientation shows a significant correlation with physical activity adherence among junior high school students (*P*<0.05). (Refer to Table 1)

**Table 1:** Correlation analysis of motivational climate, achievement goal orientation and physical activity adherence of junior high school students.

	1	2	3	4	5	6	7	8	9	10
Motivational climate	1									
Achievement climate	0.250*	1								
Mastery climate	0.879**	0.533**	1							
Physical activity adherence	0.674**	0.585**	0.596**	1						
Behavioral habits	0.503**	0.455**	0.426**	0.821**	1					
Effort investment	0.637**	0.518**	0.596**	0.879**	0.600**	1				
Emotional experience	0.555**	0.504**	0.468**	0.836**	0.551**	0.574**	1			
Achievement goal orientation	0.607**	0.531**	0.531**	0.659**	0.475**	0.559**	0.631**	1		
Task goal orientation	0.494**	0.424**	0.442**	0.554**	0.406**	0.470**	0.524**	0.908**	1	
Ego goal orientation	0.593**	0.530**	0.508**	0.623**	0.442**	0.528**	0.603**	0.866**	0.575**	1
*M*	38.440	20.390	18.050	48.460	14.170	16.460	17.830	34.910	18.180	16.730
*SD*	12.231	7.086	6.883	12.334	4.094	5.611	4.833	13.250	8.114	6.801

Note

* indicates significant correlation

** indicates highly significant correlation

*** indicates extremely significant correlation. (The same below)

#### 3.2.2 Comparison of variables between groups and within groups before and after experimental intervention

To analyze the conditions of the mastery climate group and the achievement climate group in terms of motivational climate, physical activity adherence, and achievement goal orientation, a pretest of the participants’ data was conducted before the formal experimental intervention. The results revealed no differences between the two groups in each variable dimension (*P*>0.05), indicating that, before the experimental intervention, both groups of participants were at the same level in terms of motivational climate, physical activity adherence, and achievement goal orientation. In order to validate the influence of motivational climate on the physical activity adherence and achievement goal orientation among junior high school students, a post-test of the motivational climate, physical activity adherence, and achievement goal orientation data of different experimental groups of junior high school students was conducted after the experimental intervention. The results indicated significant differences in the post-test variables for the participants in different climate experimental groups (*P*<0.05). The scores of the mastery climate group’s junior high school students in various dimensions of physical activity adherence were higher than those of the achievement climate group. This suggests that a mastery motivational climate in physical education classes is more effective in enhancing students’ physical activity adherence. Furthermore, while statistical significance of the results does not necessarily indicate the practical significance of the independent variable intervention, nor does it indicate the magnitude of the independent variable’s effect, the study utilized effect size to measure the practical significance of the motivational climate intervention. In this context, an effect size of *d* ≥ 0.2 is considered low, ≥ 0.5 moderate, and ≥ 0.8 high [13]. Following the experimental intervention, it was observed that significant changes occurred in individuals’ motivational climate, achievement goal orientation, and physical activity adherence. (Refer to Table 3)

Comparison within different groups regarding motivational climate, physical activity adherence, and achievement goal orientation reveals that the post-test scores for both the achievement climate group and the mastery climate group were higher than the pretest scores. There were extremely significant differences in the pretest and post-test scores of the different experimental groups (*P*<0.001), Looking at the mean values, it was found that the total score and scores in various dimensions of physical activity adherence for the junior high school students in the mastery climate group were higher than those in the achievement climate group. This indicates that different physical education classroom climates have a certain effect on enhancing junior high school students’ physical activity adherence, with the mastery climate in the physical education classroom demonstrating a better effect in enhancing junior high school students’ physical activity adherence. Comparing the various dimensions of achievement goal orientation within junior high school students’ groups, it was observed that the post-test scores for the total achievement goal orientation and its various dimensions in the mastery climate experimental group and the achievement climate experimental group showed significant differences (*P*<0.05). The post-test for the junior high school students in the mastery climate group tended to favor task goal orientation, whereas the choice of achievement goal orientation for the junior high school students in the achievement climate group tended to lean towards ego goal orientation. This indicates that the different motivational climates in the physical education classroom effectively alter individuals’ achievement goal orientation. (Refer to Table 4)

#### 3.2.3 Effectiveness test of motivational climate activation

To examine the perception level of junior high school students in different experimental groups regarding the creation of a motivational climate and the success of the motivational climate activation, an effect size test was utilized to assess the effectiveness of the motivational climate teaching intervention [14]. The results of the post-test data between groups indicated a Cohen’s d effect size of 0.637 for the “achievement” climate, and 0.494 for the “mastery” climate. According to the effect size standards proposed by Cohen, it can be demonstrated that the teaching intervention designed in this study successfully created an “achievement” motivational climate in the achievement group and a “mastery” motivational climate in the mastery group. Furthermore, from the perspective of the effect size Cohen’s d, it is observed that the pretest and post-test data within different experimental groups displayed significant differences, indicating that this experiment’s intervention had a positive effect on the participants’ motivational climate, physical activity adherence, and achievement goal orientation. (Refer to Tables 2–4)

**Table 2:** Between-group comparisons of dimensions in the experimental group before the experimental intervention (*n* = 82).

Variables	Dimensions	Mastery climate group	Achievement climate group	*t*	*p*
		(*n* = 42)	(*n* = 40)		
		*M±SD*	*M±SD*		
Motivational climate	Achievement climate	13.19±4.41	14.30±6.61	-0.897	0.372
	Mastery climate	16.55±6.69	14.43±5.04	1.616	0.110
Physical activity adherence	Effort investment	14.05±5.27	12.10±5.68	1.61	0.111
	Emotional experience	14.83±5.42	13.68±5.21	0.985	0.328
	Behavioral habits	11.26±4.31	10.28±3.59	1.122	0.265
Achievement goal orientation	Achievement goal orientation	12.67±6.57	12.45±7.64	0.138	0.891
	Achievement goal orientation	11.43±6.64	11.48±6.18	-0.033	0.974

**Table 3:** Between-group comparisons and post-test effect sizes for each dimension in the experimental group after the experimental intervention (*n* = 82).

Variables	Dimensions	Mastery climate group (n = 42)	Achievement climate group (*n* = 40)	*t*	*p*	Effect size of post-test Cohen’d
		*M±SD*	*M±SD*			
Motivational climate	Achievement climate	16.43±6.72	19.75±6.72	-2.237	0.028*	0.637
	Mastery climate	22.50±6.08	18.17±7.46	2.87	0.005**	0.494
Physical activity adherence	Effort investment	15.36±3.43	12.93±4.40	2.275	0.026*	0.504
	Emotional experience	17.81±5.07	15.05±5.87	2.653	0.010*	0.588
	Behavioral habits	19.17±4.32	16.43±5.00	2.783	0.007**	0.619
Achievement goal orientation	Task goal orientation	20.36±7.64	15.90±8.05	2.568	0.012*	0.568
	Ego goal orientation	18.50±5.62	14.88±7.48	2.472	0.016*	0.550

**Table 4:** Intra-group comparison of variables in experimental groups with different motivational climates before and after experimental intervention (*n* = 82).

Variables	Groups(*n*)	Pre-testing	After-testing	*t*	*P*	Cohen’d
		*M±SD*	*M±SD*			
Motivational climate	Achievement climate group (40)	28.73±11.22	37.92±13.30	-4.195	<0.001***	0.663
	Mastery climate group (42)	29.74±8.85	38.92±11.25	-6.784	<0.001***	1.047
Mastery climate	Achievement climate group (40)	14.43±5.04	18.17±7.45	-3.121	0.003**	0.494
	Mastery climate group (42)	16.55±6.69	22.50±6.07	-6.210	<0.001***	0.958
Achievement climate	Achievement climate group (40)	14.30±6.61	19.75±6.72	-4.731	<0.001***	0.748
	Mastery climate group (42)	13.19±4.41	16.42±6.71	-3.584	<0.001***	0.533
Physical activity adherence	Achievement climate group (40)	36.05±12.38	44.4±13.49	-4.577	<0.001***	0.724
	Mastery climate group (42)	40.14±11.90	52.33±9.78	-5.416	<0.001***	0.836
Effort investment	Achievement climate group (40)	12.10±5.68	15.05±5.87	-4.084	<0.001***	0.646
	Mastery climate group (42)	14.05±5.27	17.81±5.07	-3.302	0.002**	0.509
Emotional experience	Achievement climate group (40)	13.68±5.21	16.43±5.00	-3.215	0.003**	0.508
	Mastery climate group (42)	14.83±5.42	19.16±4.31	-4.958	<0.001***	0.765
Behavioral habits	Achievement climate group (40)	10.28±3.59	12.93±4.40	-4.147	<0.001***	0.656
	Mastery climate group (42)	11.26±4.31	15.36±3.43	-5.469	<0.001***	0.844
Achievement goal orientation	Achievement climate group (40)	23.93±12.38	30.78±13.15	-3.091	0.004**	0.489
	Mastery climate group (42)	24.10±12.57	38.85±12.23	-6.240	<0.001***	0.963
Task goal orientation	Achievement climate group (40)	12.45±7.63	15.90±8.05	-3.205	0.003**	0.507
	Mastery climate group (42)	12.67±6.57	20.35±7.64	-5.205	<0.001***	0.803
Ego goal orientation	Achievement climate group (40)	11.48±6.17	14.88±7.48	-2.404	0.021*	0.380
	Mastery climate group (42)	11.43±6.64	18.50±5.62	-6.703	<0.001***	1.034

### 3.3 Mediation model testing for achievement goal orientation

Based on the results of the correlation analysis, it is evident that all variables have significant pairwise correlations, meeting the fundamental requirements for constructing a mediation model. Therefore, we can proceed with the examination of the mediating effect of achievement goal orientation. The study tested the mediating effects of the collected data on motivational climate, achievement goal orientation, and physical activity adherence.

**Fig 1:**
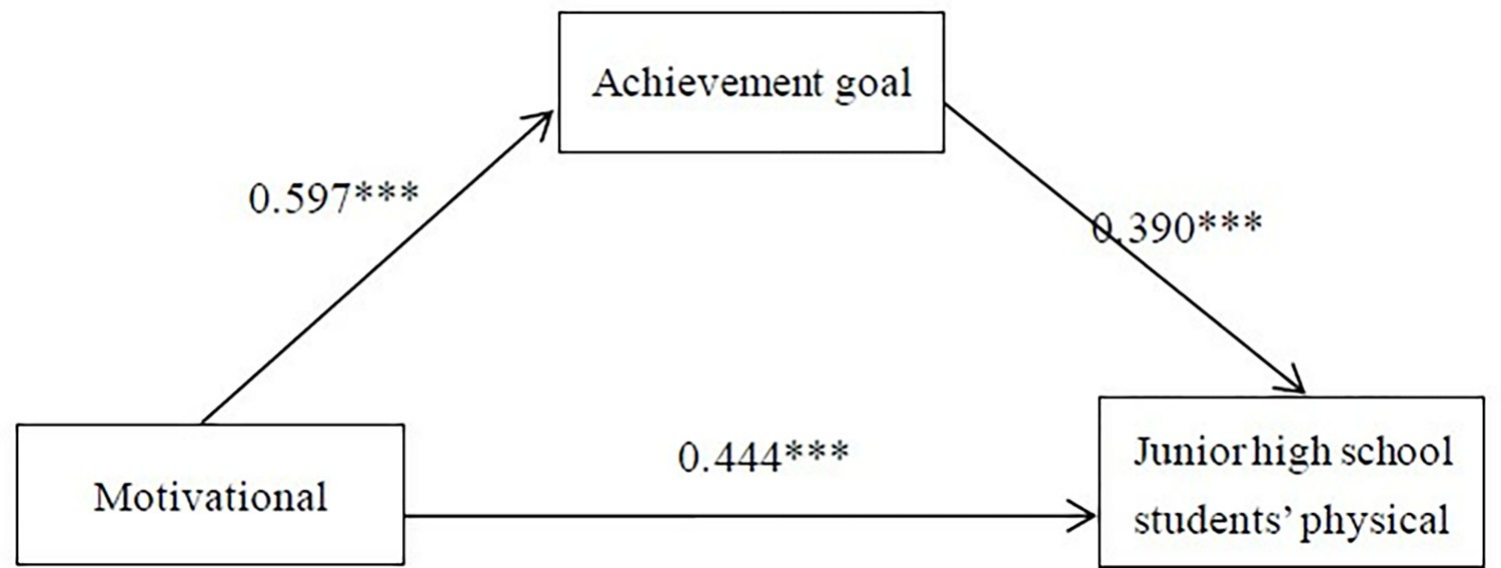
Mediation model for achievement goal orientation.

In the regression results of the mediation model testing, Model 1 represents the regression model of control variables and motivational climate on the physical activity adherence among junior high school students. The model’s goodness of fit, indicated by R-squared (R2) of 0.472 and an F-value of 23.267 (*p*<0.001), suggests a well-fitted model. The regression results reveal a positive relationship between the motivational climate and the physical activity adherence (β = 0.676, *p*<0.001). Model 2, building upon the motivational climate, incorporates the analysis of the impact of both variables on the physical activity adherence, with the addition of achievement goal orientation. The model exhibits a good fit with an R2 of 0.564 and an F-value of 24.904 (*p*<0.001). Notably, the mediation variable demonstrates a significant positive impact on the dependent variable, as achievement goal orientation positively influences the physical activity adherence (β = 0.390, *p*<0.001). Finally, Model 3 represents the influence of the mastery climate on achievement goal orientation. With an R2 of 0.396 and an F-value of 17.033 (*p*<0.001), the model shows a well-fitted goodness of fit. The results indicate a significant positive influence of the motivational climate on achievement goal orientation (β = 0.597, *p*<0.001). (Refer to Table 5 and Fig 1)

**Table 5:** Regression results of mediation model.

Model	Physical activity adherence		Physical activity adherence		Achievement goal orientation	
	*β*	*t*	*β*	*t*	*β*	*t*
Age	0.063	0.758	0.087	1.142	-0.146	-1.643
Gender	-0.125	-1.501	-0.068	-0.877	-0.062	-0.694
Motivational climate	0.677	8.185***	0.444	4.663***	0.597	6.751***
Achievement goal orientation			0.390	4.026***		
*R* ^*2*^	0.472		0.564		0.396	
*F*	23.267***		24.904***		17.033***	

The preceding analysis examined the associations between motivational climate, achievement goal orientation, and the physical activity adherence among junior high school students. In order to further investigate the mediating role of achievement goal orientation, this study, following the methods outlined by Hayes and Wen employed the bootstrap method with a 95% confidence interval and 5000 iterations. Utilizing Model 4 from the Process 4.2 plugin in SPSS 27.0, the study tested the mediating effect of achievement goal orientation between the motivational climate and the physical activity adherence. The results revealed that the direct effect accounted for 65.58% with a coefficient of 0.444, while the mediated effect represented 34.42% with a coefficient of 0.233. Importantly, the confidence interval did not include 0, indicating a significant mediated effect and thus the presence of mediation [15, 16]. (Refer to Table 6)

**Table 6:** Breakdown table of indirect effect, direct effect and total effect test results of the mediation model.

	Effect value	Boot SE	Boot CI upper limit	Boot CI lower limit	Effect ratio
Direct effect	0.444	0.123	0.194	0.676	65.58%
Indirect effect	0.233	0.083	0.091	0.415	34.42%
Total effect	0.677	0.090	0.491	0.846	/

Note: Boot standard error, Boot CI lower limit, and Boot CI upper limit respectively refer to the standard error, lower limit, and upper limit of the 95% confidence interval estimated using bias-corrected percentile Bootstrap method for the indirect effects.

### 3.4 Examination of effect of different durations on the physical activity

The effect of different durations on the physical activity adherence was examined using a repeated measures analysis of variance. Measurements were taken in the 4th, 8th, and 12th weeks. Based on the total scores of physical activity adherence, the analysis revealed an *F*-value of 20.409 and a *P*-value < 0.001. This indicates significant differences in physical activity adherence among different groups of participants at different time intervals. The results of the mean values indicate an increasing trend in the participants’ physical activity adherence as the experimental intervention progresses. (Refer to Table 7)

**Table 7:** Comparison of physical activity adherence and various dimensions at different time points.

Variables	Before intervention	The 4th week	The 8th week	The 12th week	*F*	*P*	*η* ^2^
	*M±SD*	*M±SD*	*M±SD*	*M±SD*			
Physical activity adherence	38.15±12.23	38.87±11.26	41.93±10.81	45.73±10.45	20.409	<0.001***	0.203
Effort investment	13.09±5.53	13.43±5.80	14.51±4.90	15.77±5.57	9.029	<0.001***	0.101
Emotional experience	14.27±5.32	14.41±5.08	15.54±5.66	16.72±6.32	7.734	0.002**	0.088
Behavioral habits	10.78±3.99	11.02±3.87	11.87±4.15	13.24±4.01	9.572	<0.001***	0.107

## 4. Discussion

### 4.1 The influence of different climates on physical activity adherence in junior high school students

Sports psychology highlights that motivation is the psychological impetus or internal driving force that stimulates and sustains an individual’s engagement in physical activities toward a particular goal. Motivational climate, based on different classification criteria, is considered to be an environmental factor that influences an individual’s internal motivation [17]. This climate can be established not only in physical activity but also in the context of physical education classes. Throughout the educational process, instructional methods centered around cooperation and motivation can be employed to create a motivational climate in the physical education classroom, thereby enhancing students’ adherence to physical activities [5]. According to the motivational climate theory proposed by Ames, motivational climate can be categorized into mastery climate and performance climate. These different motivational climates exert distinct influences on individuals. In a mastery climate classroom environment, the main emphasis is on engaging students to focus on the learning tasks themselves, highlighting the importance of self-comparison and effort, and emphasizing the degree of personal skill improvement. On the other hand, in a performance climate classroom environment, students’ attention is directed toward self-comparison with others, including comparisons related to learning abilities, technical skills, and academic achievements. In research on the relationship between motivational climate and physical education practice, it has been observed that different motivational climates in physical education classes are associated with the physical education learning interests of junior high school students. By creating different motivational climates in physical education classes, it has been found that both types of climates effectively improve students’ interest in physical education. However, the mastery climate has a more widespread impact on the positive psychological effects of junior high school students [18]. Regarding the influence of different motivational climates on physical activity adherence, studies have shown that the creation of a mastery climate in the classroom is more effective in enhancing college students’ adherence to physical activity compared to a performance climate in the physical education environment [19].

The results of this study indicate that both mastery climate and performance climate have positively impacted the physical activity adherence of junior high school students. However, the influence of the mastery climate on physical activity adherence is higher than that of the performance climate, aligning with the findings of some scholars. This may be attributed to the emphasis placed on creating different classroom atmospheres in physical education classes through the use of various instructional and organizational methods. The mastery climate group underscores cooperation, while the performance climate group emphasizes competition. Moreover, in terms of instructional guidance, a more straightforward and comprehensible mode of expression was employed, enabling students to more clearly perceive the classroom atmosphere in which they are situated and facilitating the enhancement of internal motivation for engaging in physical activity.

### 4.2 The mediating role of achievement goal orientation between motivational climate and physical activity adherence in junior high school students

Achievement goal orientation can be divided into task orientation and ego orientation, and the inclination towards a particular goal orientation is determined by both individual personality traits and situational factors [20]. The classroom context can influence individuals’ choice of goal orientation. Previous research has demonstrated that if the environment emphasizes mastery and learning, individuals are more likely to adopt a task orientation. On the other hand, if the environment emphasizes competition or reflects individuals’ ability levels, individuals in that context are more likely to adopt an ego orientation. Different motivational climates can significantly predict individuals’ achievement goal orientation. Furthermore, motivational climate can also predict the stability and extendibility of individuals’ achievement goal orientation. It is one of the factors influencing individuals’ achievement goal orientation. Mastery climate can predict task orientation, while performance climate can predict ego orientation [21]. On the other hand, in the studies on achievement goal orientation and physical activity adherence, it has been found that individuals inclined towards task goal orientation are more likely to accept challenging tasks and exhibit more persistence in their behavior. On the contrary, individuals inclined towards ego goal orientation tend to focus on improving themselves in comparison with others. To some extent, ego goal orientation has a lower impact on improving physical activity adherence compared to task goal orientation [8]. This suggests that individuals can change their achievement goal orientation under different motivational climates and thereby improve their physical activity adherence.

The results of this study indicate that achievement goal orientation partially mediates the relationship between motivational climate and physical activity adherence in junior high school students. Individual physical activity adherence is influenced by various external and internal factors. Motivational theory and achievement goal orientation theory are important internal factors for improving individual physical activity adherence. Additionally, the motivational climate created in physical education classes influences the outcome of individuals’ achievement goal orientation. In a mastery climate environment, individuals are more inclined to adopt a task orientation, which can improve their motivation to engage in physical activity and enhance their physical activity adherence to some extent. On the other hand, in a performance climate environment, individuals are more inclined to adopt an ego orientation, focusing on competition and comparison with others. In this type of climate, the improvement in physical activity adherence is relatively lower compared to individuals with a task orientation.

### 4.3 The improvement effects of different durations on physical activity adherence

Motivation theory suggests that the emergence and maintenance of motivation is contingent upon an individual’s internal drive and incentives. The occurrence and sustenance of physical activity adherence are closely tied to motivation. The roles of internal drive and incentives in individual motivation are distinct. Internal drive emphasizes the internal motivational force of the individual, while incentives underscore the environmental influence on motivation [22]. As external behavioral manifestations, physical activity adherence is concurrently influenced by environmental and internal factors. Therefore, in examining the factors influencing physical activity adherence, it is imperative to consider both environmental and individual factors. The motivational climate in physical education classrooms and individual achievement goal orientation, serving as incentives and internal driving forces for enhancing junior high school students’ physical activity adherence, have been confirmed in this study. In existing research on motivational climate, the intervention durations and frequencies utilized by numerous scholars are not uniform. Moreover, previous studies have not explored the changes in physical activity adherence under different intervention durations. Thus, in this study, measurements of physical activity adherence were conducted in the fourth, eighth, and twelfth weeks of formal experimental intervention. It was observed that with increasing intervention duration, the level of physical activity adherence among junior high school students improved. From the mean values, significant differences in physical activity adherence between the fourth and twelfth weeks were evident. This indicates that the improvement effects on physical activity adherence continue to increase with longer intervention periods.

### 4.4 Limitations and outlook

This study designed a motivational climate intervention program in physical education classrooms based on previous theoretical foundations and research findings. It also examined the mediating role of achievement goal orientation between motivational climate and physical activity adherence. However, there are certain limitations that should be acknowledged: Firstly, the environmental focus of this study primarily revolved around physical education classes. Future research could apply motivational climate to different sports groups and sports environments, such as athletes from different disciplines and recreational sports. Secondly, the study primarily relied on student evaluations to measure the motivational climate created by physical education teachers and students’ physical activity adherence. Future research could utilize methods such as parental evaluations, peer evaluations, and teacher self-assessments to examine the research variables. Thirdly, this study adopted a cross-sectional design, making it difficult to establish causal relationships between the research variables. Future research could employ intervention studies to further investigate the causal relationships among the variables.

## 5. Conclusions

The results of the study showed that the physical education classroom motivational climate created during the experimental intervention significantly and positively predicted junior high school students’ physical activity adherence and achievement goal orientations, with achievement goal orientations playing a partially mediating role between the physical education classroom motivational climate and junior high school students’ physical activity adherence. The results of the experimental intervention showed that the effect of improving the adherence to physical activity of junior high school students in the mastery atmosphere experimental group was higher than that of junior high school students in the achievement atmosphere experimental group; at the same time, the effect of improving the adherence to physical activity will be improved with the increase in the length of the intervention, and the effect of the twelve-week intervention in improving the adherence of junior high school students to physical activity was better than that of the four-week and eight-week intervention. The findings of this study can enrich the research results on the correlation between motivational climate, achievement goal orientation and physical activity adherence, and provide a certain theoretical basis and practical reference for school sports to improve students’ physical activity adherence, as well as for physical education teachers to effectively cultivate students’ physical activity adherence behaviours in future teaching. Future research could design different classroom motivational climate building programmes for different sports and teaching methods to enhance the relevance and completeness of classroom motivational climate building programmes.

## Supporting information

S1 Appendix Questionnaire. (DOCX)
