# Supplementary material for: The influence of motivational climate on the physical activity adherence among junior high school students: The mediating effect of achievement goal orientation
Source: PLoS One. 2024 Dec 19;19(12):e0315831. doi: 10.1371/journal.pone.0315831 (PMC11658482; doi:10.1371/journal.pone.0315831)
Supplement: S1 Appendix — (DOCX) [file pone.0315831.s001.docx]

**Appendix 1: Questionnaire**

**Motivational Climate Scale (MCSYS)**

| **Item** | **Statement** | **Completely Inconsistent** | **Somewhat Inconsistent** | **Neutral** | **Somewhat Consistent** | **Completely Consistent** |
| --- | --- | --- | --- | --- | --- | --- |
| 1 | According to the physical education teacher, winning a game is the most crucial factor. |  |  |  |  |  |
| 2 | The physical education teacher ensures that students enjoy themselves while enhancing their skills. |  |  |  |  |  |
| 3 | The physical education teacher invests minimal time with students who exhibit poor performance. |  |  |  |  |  |
| 4 | The physical education teacher motivates us to acquire new skills. |  |  |  |  |  |
| 5 | The physical education teacher enlightens us about the most outstanding students. |  |  |  |  |  |
| 6 | The physical education teacher emphasizes mutual support and collective progress. |  |  |  |  |  |
| 7 | The physical education teacher stresses that giving our utmost effort is paramount. |  |  |  |  |  |
| 8 | The physical education teacher places considerable attention on students displaying outstanding performance. |  |  |  |  |  |
| 9 | The physical education teacher frequently advocates that students should enhance their skills through mutual assistance. |  |  |  |  |  |
| 10 | If a student errs, they will face reprimand from the physical education teacher. |  |  |  |  |  |
| 11 | The physical education teacher asserts that each student is indispensable for the team’s success. |  |  |  |  |  |
| 12 | The physical education teacher encourages us to endeavor surpassing our classmates. |  |  |  |  |  |

**Task and Ego Orientation in Sport Questionnaire (TEOSQ)**

| **Items** | **In sport, I feel most successful when...** | **Completely Inconsistent** | **Somewhat Inconsistent** | **Uncertain** | **Somewhat Consistent** | **Completely Consistent** |
| --- | --- | --- | --- | --- | --- | --- |
| 1 | I learn a new technique and it makes me want to practice more. |  |  |  |  |  |
| 2 | I am the only one who has mastered a particular technique or skill. |  |  |  |  |  |
| 3 | I learn a movement that I find enjoyable. |  |  |  |  |  |
| 4 | I outperform my classmates. |  |  |  |  |  |
| 5 | I work hard to learn a new technique. |  |  |  |  |  |
| 6 | Others cannot perform as well as I can. |  |  |  |  |  |
| 7 | I train very hard. |  |  |  |  |  |
| 8 | Others have problems while I do not. |  |  |  |  |  |
| 9 | I learn a technique that motivates me to practice more. |  |  |  |  |  |
| 10 | I achieve the best or highest score. |  |  |  |  |  |
| 11 | A newly learned movement is mastered well. |  |  |  |  |  |
| 12 | I am the best performer. |  |  |  |  |  |
| 13 | I give my maximum effort. |  |  |  |  |  |

**Physical Activity Adherence Scale**

| **Item** | **Statement** | **Completely Disagree** | **Somewhat Disagree** | **Neutral** | **Somewhat Agree** | **Completely Agree** |
| --- | --- | --- | --- | --- | --- | --- |
| 1 | I engage in physical activities for at least an hour each time. |  |  |  |  |  |
| 2 | I have been exercising regularly for at least the past 6 months. |  |  |  |  |  |
| 3 | I participate in physical activity at least three times a week. |  |  |  |  |  |
| 4 | I have developed the habit of exercising when it is time to do so. |  |  |  |  |  |
| 5 | I strive to learn new skills to enhance my abilities. |  |  |  |  |  |
| 6 | Regardless of my enjoyment, I exert maximum effort every time I engage in physical activity. |  |  |  |  |  |
| 7 | I am determined to maintain a consistent physical activity regimen. |  |  |  |  |  |
| 8 | I am willing to allocate a fixed portion of my schedule to ensure regular physical activity. |  |  |  |  |  |
| 9 | I make an effort to eliminate distractions to adhere to my physical activity routine. |  |  |  |  |  |
| 10 | I appreciate the sensation brought about by physical activity. |  |  |  |  |  |
| 11 | I enjoy exercising with others. |  |  |  |  |  |
| 12 | I often feel uplifted after exercising. |  |  |  |  |  |
| 13 | I often feel strengthened after exercising. |  |  |  |  |  |
| 14 | I experience a sense of physical and mental well-being after exercising. |  |  |  |  |  |
